# Causal chain event graphs for remedial maintenance

**DOI:** 10.1111/risa.14308

**Published:** 2024-04-23

**Authors:** Xuewen Yu, Jim Q. Smith

**Affiliations:** ^1^ Statistics department University of Warwick Coventry UK; ^2^ The Alan Turing Institute London UK

**Keywords:** artificial intelligence, causal identification, chain event graphs, reliability analysis

## Abstract

The analysis of system reliability has often benefited from graphical tools such as fault trees and Bayesian networks. In this article, instead of conventional graphical tools, we apply a probabilistic graphical model called the chain event graph (CEG) to represent the failures and processes of deterioration of a system. The CEG is derived from an event tree and can flexibly represent the unfolding of asymmetric processes. For this application, we need to define a new class of formal intervention we call remedial to model the causal effects of remedial maintenance. This fixes the root causes of a failure and returns the status of the system to as good as new. We demonstrate that the semantics of the CEG are rich enough to express this novel type of intervention. Furthermore, through the bespoke causal algebras, the CEG provides a transparent framework with which to guide and express the rationale behind predictive inferences about the effects of various types of remedial intervention. A backdoor theorem is adapted to apply to these interventions to help discover when a system is only partially observed.

## 1 INTRODUCTION

Conventional graphical tools in system reliability include fault trees (FTs) and Boolean decision diagrams (BDDs). An FT is a structured top–down logic diagram starting with the critical system event and decomposing successively into events whose composition or intersection can cause the top event (Bedford & Cooke, 2001). A BDD can provide an equivalent graphical representation to an FT, where events are ordered in the same way as an event tree. Neither of these diagrams provides explicit partial (temporal) order of events nor standard statistical modeling methodologies associated with uncertainty handling and causal reasoning can be seamlessly embedded. Early work (Cai et al., 2018; Pasquini et al., 1999; Torres‐Toledano & Sucar, 1998) also criticized traditional reliability analysis, stating that the deficiency of these models lies in the limitation of modeling the uncertain dependencies between failures and complex systems and suggested instead the use of a Bayesian network (BN). Both BNs and chain event graphs (CEGs) enjoy the flexibility of embedding probabilistic knowledge, managing probability propagation, inference, and performing causal analysis. Thus, these two classes of models can be used to inform decision makers or engineers about the potential effects of new policies or actions, enabling optimization of the maintenance strategy in an efficient and effective way—making probabilistic graphical tools more appealing for reliability or risk analysis than traditional tools.

Despite the popularity of the BN framework for exploring causal relationships, many researchers (Riccomagno, 2005; Shafer, 1996; Spirtes et al., 2000) have argued that event tree‐based inference provides an even more flexible and expressive graph from which to explore causal relationships. Within artificial intelligence, methods based on probability trees are now widely used for various types of causal modeling to support decision and risk analyses in many different domains, see, for example, causal discovery, decision making, and risk analysis (Bedford & Cooke, 2001; Bunnin & Smith, 2021; Genewein et al., 2020; Zhao et al., 2017).

Within the domain of reliability where the focus of inference is on explaining and repairing failure incidents in a system, the use of trees and their derivative depictions such as CEGs (Collazo, 2017; Collazo et al., 2018; Riccomagno, 2005) provide a complementary method to the use of FTs. Here, a collection of paths on these graphs are used to explain the unfolding of events that might have led to the fault. One advantage of the CEG is that unlike the BN the asymmetric unfoldings of the process can be directly represented by its topology so that context‐specific causal dependencies can be read from the tree (Anderson & Smith, 2006; Barclay et al., 2013; Smith & Anderson, 2008). This is extremely useful for encoding explicitly the failure processes and deteriorating processes of machines.

Robins (1986) demonstrated and Shafer (1996) has long argued that causal assumptions are often easily inferred from tree‐like structures because these represent explicitly the hypothesized time orderings of events intrinsic to many causal conjectures. A causal analysis can be performed around a framework of the CEG in much the same way as for the BN. However, although the BN has been successfully applied to support the causal analysis of various problems in reliability (Fenton & Neil, 2018), to our knowledge the more flexible framework of the CEG has yet to be applied to this domain. We show in this article that the CEG is a much more expressive graphical representation than the BN for putative causes (Anderson & Smith, 2006; Collazo et al., 2018; Cowell & Smith, 2014; Thwaites, 2013) and it can embed the sorts of asymmetries met in reliability models yet to be exploited.

Previous work (Thwaites, 2013; Thwaites et al., 2010) has proposed a generic method to translate Pearl's *do*‐calculus (Pearl, 1995, 2009) onto the CEGs. The atomic intervention on the BN that forces a variable to take a single value can be simply imported into the CEG where a singular manipulation on a causal BN corresponding to forcing multiple *edges* along the equivalent causal CEG to take a conditional probability one and others zero. For such conventional types of manipulations, it has been discovered when these and more nuanced interventions can be identified. In particular, Thwaites (2008, 2013) and Thwaites et al. (2010) formulated the backdoor theorem and the front‐door theorem on the CEG, analogous to what Pearl (Galles & Pearl, 1995; Pearl, 1995, 2009) designed on the BNs. These theorems support predictive models regarding how certain natural events might trigger failures.

Causal reasoning about interventions to study the reliability of a system can inform the policy makers or the engineers about the potential effects of new policies or actions so that the maintenance strategy can be optimized in an efficient and effective way. However, for models of system reliability, we may encounter complicated forms of causal mechanisms and unfamiliar types of intervention not usually studied in standard causal analyses when failure events automatically trigger remedial maintenance. For example, when such remedial acts involve the replacement of failed components, they will behave as if starting from new rather than starting from a point of embedded usage as they would be in a more conventional causal intervention. The latter would be what we would need to assume were we to use conventional algebras. But perhaps even more important is that remedial interventions we study here are always designed to rectify a subset of *root causes* of a failure incident. So interventions associated with remedial maintenance are in practice nearly always very specific types of nonatomic (not singular) interventions. We demonstrate that the manipulations associated with such interventions are then likely not to be represented within the class of vanilla interventions considered in causal BNs—they are far too symmetric.

Yu et al. (2020) gave a brief introduction of different types of remedies. Here, we formalize these ideas and provide a detailed methodology which imports the concept of remedy and a root cause analysis into the CEG framework. In this way, we are about to establish new causal algebras for the different remedial intervention regimes on CEGs. We show how to use CEGs to determine when and if so how we can measure probabilistically the effectiveness of remedies imposed by engineers to perceived faults.

The contributions of this article are threefold. First, we propose a novel approach for causal analysis which is applicable to reliability data. We formalize the concept of the stochastic manipulation on the CEG and develop the mathematical formulas to import it into the CEG. Moreover, we show the causal effects of stochastic manipulations can be identified on CEGs using the adapted backdoor theorem. Both graphical criteria and proofs are given to support this new theorem. Third, we define a new type of intervention—the remedial intervention—on the CEG for analyzing system reliability. In particular, we emphasize useful causal concepts like “remedy” and “root cause” in reliability and translate them into algebras via CEGs to embellish the standard causal analysis.

In the next section, we will show how to construct a CEG for analyzing system reliability. Then using this framework to formally define a remedial intervention in Section 3. In Section 4, we apply this definition to prove a number of results about whether or not the probabilistic effects of a given intervention are identifiable from information commonly available to an engineer. Section 5 will use a simulated data set to demonstrate how to perform a causal analysis using the techniques proposed in previous sections.

## 2 CONSTRUCTING CEGS FOR RELIABILITY ANALYSIS

In this section, we will briefly review the elicitation process of CEGs from event trees and introduce new concepts for constructing a CEG for reliability data.

### 2.1 A review of CEGs

Consider a finite event tree $T=(V_{T},E_{T})$ defined with vertex set $V_{T}$ and edge set $E_{T}$ (Collazo et al., 2018; Görgen & Smith, 2018; Smith, 2010; Smith & Anderson, 2008; Thwaites, 2008). Let $e_{v,v^{\prime }}\in E_{T}$ denote the directed edge emanating from the vertex v and pointing to the vertex $v^{\prime }$. For any vertex $v\in V_{T}$, denote the set of its parents by $pa\left(v\right)=\left\{v^{\prime }\in V_{T}:e_{v^{\prime },v}\in E_{T}\right\}$, and the set of its children by $ch\left(v\right)=\left\{v^{\prime }\in V_{T}:e_{v,v^{\prime }}\in E_{T}\right\}$. The vertex without parents in T is called the *root* vertex of the tree, denoted by $v_{0}$. The set of vertices without children are the *leaves* of the tree, denoted by $L_{T}\subset V_{T}$. The nonleaf vertices are called the *situations* here, denoted by $S_{T}=V_{T}\setminus L_{T}$. A *floret* $F\left(v\right)=\left\{V_{F(v)},E_{F(v)}\right\}$ of a situation $v\in V_{T}$ is a subtree of T, whose vertex set $V_{F(v)}$ consists of v and ch(v), and whose edge set is $E_{F(v)}=\left\{e_{v,v^{\prime }}\in E_{T}:v^{\prime }\in ch\left(v\right)\right\}$. A path starting from $v_{0}$ and terminating in $v\in L_{T}$ is called a *root‐to‐leaf path* on the tree. Let $\Lambda_{T}$ denote the collection of all the root‐to‐leaf paths of T.

If each edge $e_{v,v^{\prime }}\in E_{T}$ has an associated transition probability, denoted by $\theta_{v,v^{\prime }}$, such that $\sum_{v^{\prime }\in ch\left(v\right)}\theta_{v,v^{\prime }}=1$ and $\theta_{v,v^{\prime }}\in \left(0,1\right)$, then a *probability tree* with structure T and set of probabilities $\theta_{T}={\left\{\theta_{v}\right\}}_{v\in S_{T}}$ can be well‐defined. The probability vector $\theta_{v}={\left(\theta_{v,v^{\prime }}\right)}_{v^{\prime }\in ch\left(v\right)}$ is defined for each situation. Then, the probability of traversing along any path can be evaluated. Let π(·) represent the path related probability on the tree and $\theta_{v,v^{\prime }}=\pi \left(v^{\prime }|v\right)$.

A *staged tree* (Collazo et al., 2018) is a colored probability tree $\left(T,\theta_{T}\right)$ where different color represent different *stages*. Two situations $v,v^{\prime }$ are in the same stage if the probability distributions over the set of edges $E\left(v\right),E\left(v^{\prime }\right)$ are the same. Let $U_{T}=\left\{u_{0},\text{…},u_{n}\right\}$ denote the set of stages of T. Given a staged tree, two situations $v,v^{\prime }$ in the same stage are in the same *position* if and only if T(v) and $T(v^{\prime })$ are isomorphic, that is, having same set of edges, vertices, and coloring. Let $W_{T}=\left\{w_{0},\text{…},w_{m}\right\}$ denote the set of positions. All the leaves in the staged tree belongs a sinking status, denoted by $w_{\infty }$. A *chain event graph* can then be constructed by merging the vertices that belong to the same position and vertices in the sinking status and the corresponding edges that share the same color. Let $C=(V_{C},E_{C})$ denote the graphical representation of the CEG. The vertex set is $V_{C}=W_{T}\cup w_{\infty }$. If edges $e_{v,v^{\prime }},e_{w,w^{\prime }}^{\prime }\in E_{T}$ and v,w are in the same position, then there exist corresponding edges $f,f^{\prime }\in E_{C}$. If also $v^{\prime },w^{\prime }$ are in the same position, then $f=f^{\prime }$. The positions and edges retain their corresponding color from the staged tree. The probabilities $\theta_{f}\in \theta_{C}$ of edge $f\in E_{C}$ in the new graph are the same as the transition probabilities of the corresponding edges in the staged tree. Then, a CEG is defined as $\left(C,\theta_{C}\right)$. Let $w_{0}\in W_{T}$ denote the root node of the CEG. The path starting from $w_{0}$ and terminating in $w_{\infty }$ is the *root‐to‐sink path*. Let $\Lambda_{C}$ denote the collection of all the root‐to‐sink paths on C.

### 2.2 A CEG for reliability analysis

An event tree T is next constructed to represent how system may fail. The sequence of events which would have occurred prior to maintenance are explicitly represented on each of the root‐to‐leaf path. Call the labels of events on edges by *d‐events* and denote it by $X_{T}={⋃}_{e\in E_{T}}x\left(e\right)$. Leaves represent the final status of the system, states are labeled by fail or not fail. We add restrictions to the definition of stages: v and $v^{\prime }$ are in the same stage if and only if E(v) and $E(v^{\prime })$ represent the same set of d‐events and $\theta_{v,v^{\prime }}=\theta_{v^{*},v^{{*}^{\prime }}}$ if $x\left(e_{v,v^{\prime }}\right)=x\left(e_{v^{*},v^{{*}^{\prime }}}\right)$. Unusually for this application of CEGs, it is useful to define two sink nodes for C: if $v\in L_{T}$ represents a failed condition, then $v\in w_{\infty }^{f}$, otherwise $v\in w_{\infty }^{n}$ represents an operational but worn‐out condition. We call these the *failure* and the working sink nodes, respectively. Thus, the vertex set of the CEG is $V_{C}=W_{T}\cup w_{\infty }^{f}\cup w_{\infty }^{n}$. For any path $\lambda \in \Lambda_{C}$ that ends in the sink $w_{\infty }^{f}$, we call a *failure path* and represents a possible pathway to fail. All other paths—those which terminate in $w_{\infty }^{n}$ are called *deteriorating paths*. Let $\Lambda_{C}^{f}$ and $\Lambda_{C}^{n}$ denote the sets of failure paths and deteriorating paths, respectively, so that $\Lambda_{C}=\Lambda_{C}^{f}\cup \Lambda_{C}^{n}$ and $\Lambda_{C}^{f}\cap \Lambda_{C}^{n}=\emptyset$.

CEGs have the advantage over BNs to explicitly expressing within its topology the pathway to failure. More explicitly, the chronological development of the failure or deteriorating processes can be captured by the root‐to‐sink paths. We can order the d‐events by beginning with root causes, followed by the cascading events initiated by the root causes, that is, primary faults and secondary faults and so on, and ending with a failure or worn‐out condition. Because a cause will always happen before an effect the order of these cascading events, expressed in the CEG, embodies the full causal story about what happens if a unit passes along one of its paths. In particular, this enables us to infer the nature of a root cause. In this way, we can use the topology of the CEG needed to examine the effects of a given remedial act in fixing the root cause of a failure.

**FIGURE 1:**
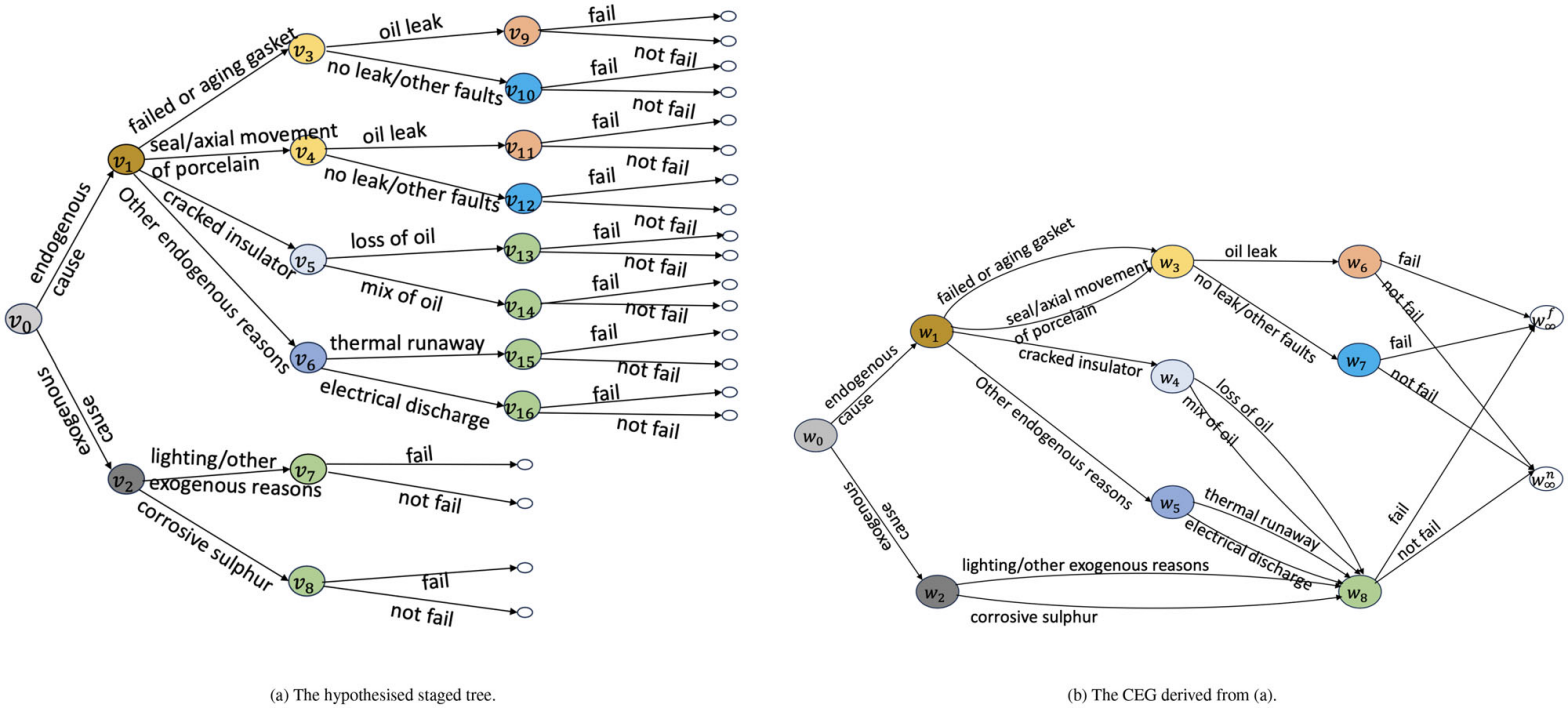
An example of the staged tree and the chain event graph (CEG) derived for a bushing system.

To demonstrate how such a CEG analysis begins, consider an example of failures associated with bushing systems depicted in Figure 1B. Bushings are components in a transformer for insulation. We constructed an event tree according to the investigation report by Al Abri et al. (2017). The tree starts with classification of causes that may lead to system failures. This is followed by depicting the potential causes, then the symptoms that might arise from these. The last component represented on the tree is the failure indicator. A staged tree next colors the vertices of T to embed various context‐specific conditional independences that an engineer might bring to the study. For example, here we assume that when the environment outside the machine impacts the system negatively, system failure is conditionally independent of the exact exogenous environment. This assumption makes situations $v_{7}$ and $v_{8}$ in the same stage. The stages in Figure 1A are $u_{0}=\left\{v_{0}\right\},u_{1}=\left\{v_{1}\right\},u_{2}=\left\{v_{2}\right\},u_{3}=\left\{v_{3},v_{4}\right\},u_{4}=\left\{v_{5}\right\},u_{5}=\left\{v_{6}\right\},u_{6}=\left\{v_{7},v_{8},v_{13},v_{14},v_{15},v_{16}\right\},u_{7}=\left\{v_{9},v_{11}\right\},u_{6}=\left\{v_{10},v_{12}\right\}$. Figure 1B presents the CEG derived from this staged tree. The positions are $w_{0}=\left\{v_{0}\right\}$, $w_{1}=\left\{v_{1}\right\}$, $w_{2}=\left\{v_{2}\right\}$, $w_{3}=\left\{v_{3},v_{4}\right\},w_{4}=\left\{v_{5}\right\},w_{5}=\left\{v_{6}\right\}$, $w_{6}=\left\{v_{9},v_{11}\right\},w_{7}=\left\{v_{10},v_{12}\right\},w_{8}=\left\{v_{7},v_{8},v_{13},v_{14},v_{15},v_{16}\right\}$.

In practice, we can construct an event tree for a system using domain knowledge, and elicit the CEG from it based on expert judgment or making appropriate assumptions. If there are data available for this system, we can apply the structural learning algorithm (Cowell & Smith, 2014; Collazo et al., 2018) to find the topology of the staged tree that best describes the data and derive the CEG accordingly: just as we would if modeling with a BN.

We can now perform causal analysis on a CEG through extending Pearl's *do‐*operation (Pearl, 2009). This is defined as the *singular manipulation* (Thwaites, 2008, 2013; Thwaites et al., 2010). Thwaites and others (Barclay, 2014; Thwaites et al., 2010) formalized a *causal CEG* as follows: under a singular manipulation at a position w so that the event represented on $e_{w,w^{*}}$ is controlled, the CEG is a manipulated CEG with $\theta_{w,w^{*}}=1$, $\theta_{w,w^{\prime }}=0$ for $w^{\prime }\in ch\left(w\right),w^{\prime }\neq w^{*}$, and all other $\theta_{w^{\prime \prime }}$, $w^{\prime \prime }\in W_{T},w^{\prime \prime }\neq w$, are unchanged. Notice that because the different root‐to‐leaf paths of the underlying event tree are expressed explicitly within the CEG, it is possible to express explicitly within its topology a much wider range of interventions than would ever be possible just using a BN. It is this property which enables us to develop a transparent causal algebra which is particularly suited to support the study of the causes of failure in system reliability.

## 3 CAUSAL ALGEBRAS FOR THE REMEDIAL INTERVENTION

The term “*remedial work*” is ubiquitous to many types of engineering reports which record the maintenance of some defects or failures. Furthermore, “*remedy*” is a more familiar terminology in reliability engineering than “treatment” (Rubin, 2004), which is commonly adopted in medical science and has a subtle different meaning. A unit must have been failed before a remedy is applied. The remedy aims to find and fix the root cause of the observed failure in order to prevent the same defect or failure reoccurring. In contrast, a treatment can be applied irrespective of the state of the unit. Furthermore, while a remedy could be a single act it is often a combination of acts taken in sequence. So the application of causal analyses are rather different in system reliability than in medicine and public health where the majority of causal analyses have traditionally been applied.

In light of the two essential concepts, that is, the remedy and the root causes, we define a novel domain‐specific intervention and call it the *remedial intervention*. This is a typical external intervention customized to different types of remedies. The inferential framework of the remedial intervention focuses on the discovery of root causes of a fault and the identification of a sequence of actions that will provide a remedy to that fault. Analogously to the root cause analysis, this process can be used to understand and prevent defects of a system by tracing and correcting the initial contributing factor of these defects. Here, we develop bespoke causal algebras on CEGs where remedial maintenance takes center stage. Such new algebras extend the singular manipulation which are now well established for CEGs (Thwaites et al., 2010; Thwaites, 2013).

### 3.1 Perfect, imperfect, and uncertain remedial interventions

For a repairable system, there are three main categories of maintenance: *perfect maintenance*, *imperfect maintenance*, and *minimal maintenance* (Borgia et al., 2009; Iung et al., 2005). If the status of the system after maintenance is the same as new and has the same failure rate, then the maintenance is *perfect* and the postmaintenance status is called *as good as new* (AGAN) (Borgia et al., 2009; Bedford & Cooke, 2001; Iung et al., 2005). If the status of the system after the maintenance returns to the working order just prior to failure, then the maintenance is *minimal*, and the postmaintenance status is called *as bad as old* (ABAO) (Borgia et al., 2009; Iung et al., 2005). If the status of the system after maintenance is somewhere between ABAO and AGAN, then the maintenance is classified to be *imperfect* (Iung et al., 2005).

To reflect these standard categories of maintenance, we accordingly give definitions to three types of remedial interventions: the *perfect remedial intervention*, the *imperfect remedial intervention*, and the *uncertain remedial intervention*. Here, we use the name “uncertain” instead of “minimal” because the causal algebras we develop later in this article concern about quantifying the characteristics of the uncertainty associated with this type of intervention.

We first make the following two assumptions before formalizing the concept of a remedial interventions.

Assumption 1 The idle CEG^1^ or the event tree is faithfully constructed with respect to the domain knowledge of a particular system so that every failure process or deteriorating process that may happen in this system can be identified on the tree and every root cause and symptom are well‐captured by the semantics of the tree.

Assumption 2 The system modeled by the CEG is repairable, and the AGAN status is reached when the root cause of the failure is completely fixed.

**FIGURE 2:**
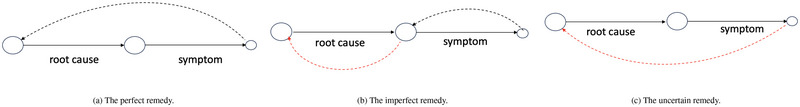
The status monitors for the three types of remedies.

For illustrative purpose, we create a new graphical framework integrating a failure process with the process of maintenance in order to demonstrate the differences between various types of the remedial intervention (see Figure 2).

Here, we simplify the root‐to‐sink path by labeling only the root cause and the symptom. Take a failure process as an example. The root vertex of this path represents an AGAN status while the sink vertex of this path represents a failed condition. We call the root vertex the AGAN vertex and the leaf vertex the fail vertex. The failure path is connected by the solid black edges. The *recovery path* is defined to be the directed dashed path rooting from the fail vertex and sinking in the AGAN vertex. It models the status change of the system caused by the maintenance. The black and red dashed edges are associated with observed and unobserved maintenance, respectively. Recovery paths are external to the idle CEG. This is because it represents the effect of external intervention on status of the equipment and such recovery process is not part of the description of the original system before any intervention has taken place.

A remedial intervention is *perfect* if the root cause of the failure is correctly identified and successfully fixed by the observed maintenance so that the postintervention status of the part being maintained is AGAN (Yu et al., 2020; Yu & Smith, 2021b). The recovery process is demonstrated in Figure 2A. The recovery path starts from the fail vertex and ends in the AGAN vertex which means the observed maintenance returns the status of the failed part to full working order. Suppose the CEG in Figure 1B faithfully models the unmanipulated bushing system, and we observe a failed bushing whose failure was caused by a cracked insulator. Then an example of a perfect remedial intervention is that the engineer replaced the cracked insulator by a new one.

If the root cause is not remedied but only a subset of the secondary or intermediate faults are remedied, then after the intervention the status of the repaired component will not return to AGAN. However, it is better than ABAO. We call such an intervention an *imperfect remedial intervention*. We can visualize the status change of the maintained equipment from Figure 2B. The recovery path consists of a black dashed edge and a red dashed edge. The black dashed edge points from the fail vertex to the interior vertex of the failure path, which means the status of the equipment is improved but not AGAN after maintenance. In order to fully restore the system, additional maintenance is needed. If imperfect remedial work has been made at time t, then the maintenance log will record only that maintenance has happened. As for what is further needed to fully restore the system is unknown at that time. This brings uncertainty into this type of remedial intervention. The recovery process corresponding to the additional remedial work is represented by the red dashed edge, which points from the interior vertex to the AGAN vertex.

If the maintenance logs do not record what remedial maintenance was taken, then such intervention is classified as an *uncertain remedial intervention*. Diagnostic information has not yet been made available so the root cause of the failure cannot be determined. A follow‐up check and maintenance will be carried out in order to restore the broken part. Therefore, the recovery process of this type of intervention is unobserved and so uncertain. The corresponding recovery path is shown in Figure 2C.

### 3.2 Notation and definitions

To model remedial intervention, we first introduce some new variables.

Assume that the root causes of a specific defect or failure could be multiple and are well‐defined. Note that a remedial intervention is defined to allow multiple root causes to be corrected simultaneously. Such an intervention is of course always nonsingular within the CEG representation.

Let A denote the state space of maintenance events. Let $A^{O}$ and $A^{U}$ be random variables taking values in A representing observed maintenance and uncertain maintenance, respectively. Let $A=(A^{O},A^{U})$ denote the vector of all maintenance. The status of the maintained equipment is observable, and represented by a *status indicator* δ such that

(1) $$\delta =\left\{\begin{array}{cc} 1, & \;\text{if the status is AGAN after maintenance,}\\ 0, & \;\text{otherwise.} \end{array}\right.$$

Let $E^{\Delta }=\left\{e_{l_{1}},\text{…},e_{l_{n}}\right\}$ denote the set of edges labeled by the d‐events associated with root causes. For any edge representing a root cause $e_{l_{i}}\in E^{\Delta }$, we define a binary variable to indicate whether or not the root cause labeled on $e_{l_{i}}$ is fixed and call it an *intervention indicator*. Let

(2) $$I_{e_{l_{i}}}=\left\{\begin{array}{cc} 1, & \;\text{if the root cause represented on}\;I_{e_{l_{i}}}\\ & \;\text{is fixed by the maintenance,}\;\\ 0, & \;\text{otherwise.} \end{array}\right.$$

Then, we have a vector of intervention indicators defined over $E^{\Delta }$, denoted by $I_{E^{\Delta }}=\left\{I_{e_{l_{1}}},\text{…},I_{e_{l_{n}}}\right\}$. Let $\lambda^{O}\in \Lambda$ be the set of possible root‐to‐sink paths associated with the failure or deterioration. Note that the whole failure or deteriorating process might be partially observed when the root causes are unknown. Let $\lambda^{R}\in \Lambda$ denote the actual failure or deteriorating path when root causes are known.

When δ=1, the remedial intervention is perfect. In other words, the root causes are correctly identified and fixed by $A^{O}$. So the value of the vector of the intervention indicators is known, denote this by $I_{A^{O}}$. Then, we have,

(3) $$p\left(I_{E^{\Delta }}|A,\lambda^{O},\delta =1\right)=\left\{\begin{array}{cc} 1, & I_{E^{\Delta }}=I_{A^{O}},\\ 0, & I_{E^{\Delta }}\neq I_{A^{O}}. \end{array}\right.$$

The actual paths $\lambda^{R}$ given $\lambda^{O}$, $A^{O}$, δ=1 are deterministic: $\lambda^{R}=E_{A^{O}}\cap \lambda^{O}$, where $e\in E_{A^{O}}$ if and only if $I_{e}=1$.

When δ=0, the remedial intervention is imperfect or uncertain. The uncertainty arises from the unobserved additional maintenance $A^{U}$. Then, the root causes need to be inferred:

(4) $$\begin{array}{cc} p(I_{E^{\Delta }}|A^{O},\lambda^{O},\delta =0)= & \sum_{a^{u}\in A}p\left(I_{E^{\Delta }}|a^{O},a^{U},\lambda^{O},\delta =0\right)\\ & p(a^{U}|a^{O},\lambda^{O},\delta =0). \end{array}$$

The probability $p\left(I_{E^{\Delta }}|a^{O},a^{U},\lambda^{O},\delta =0\right)\neq p\left(I_{E^{\Delta }}|a^{O},a^{U},\lambda^{O},\delta =1\right)$ since the latter is associated with a perfect remedy with degenerate probability distribution while the former is not. The actual path $\lambda^{R}$ can be inferred in the same way. Here, we apply Equation (4) for either imperfect or uncertain remedial intervention. This is because both types involve uncertainty in maintenance events, which is represented by the model $p(a^{U}|a^{O},\lambda^{O},\delta =0)$. When the intervention is uncertain, $a^{O}=\emptyset$ denotes an empty set and $a^{U}$ is informed from the partially observed failure process $\lambda^{O}$. In practice, we can specify a parametric model $p(a^{U}|a^{O},\lambda^{O},\delta =0;\eta_{\lambda }^{I(a^{O}\neq \emptyset )})$. Then, $\eta_{\lambda }^{0}$ will denote the set of parameters defined over the set of observable maintenance and the root‐to‐sink paths for the model under the uncertain remedial intervention, while $\eta_{\lambda }^{1}$ will denote the set of parameters defined over the root‐to‐sink paths for the model under the imperfect remedial intervention.

Thus, given the observed maintenance, we can infer the probabilities associated with the intervention indicator vector as

(5) $$\begin{array}{ccc} p(I_{E^{\Delta }}|A^{O},\lambda^{O}) & = & p\left(I_{E^{\Delta }}|\delta =1,\lambda^{O},A^{O}\right)p\left(\delta =1|\lambda^{O},A^{O}\right)\\ & & \;+\sum_{a^{u}\in A}p\left(I_{E^{\Delta }}\right|a^{O},a^{U},\lambda^{O},\\ \delta & = & 0)p\left(a^{U}|a^{O},\lambda^{O},\delta =0\right)p\left(\delta =0|\lambda^{O},A^{O}\right). \end{array}$$

Here, $p(a^{U}|a^{O},\lambda^{O},\delta =0)$ will be 0 or near 0 for rare events. Furthermore, under Assumption 1, for any system of interest, all possible root causes are represented by the tree. Under Assumption 2, Equation (5) enables us to identify the corresponding root causes for any remedial intervention. Therefore, Equation (5) provides a general form for modeling any remedial intervention.

### 3.3 The stochastic manipulation

Engineers address root causes to prevent the fault or failure caused by these root causes reoccurring. So, it is natural to assume that the distribution over root causes are affected by the remedial intervention. We then import this idea to the idle CEG. Denote the set of positions whose emanating edges are labeled by root causes as $W^{\Delta }$. A position $w\in W^{\Delta }$ if $e_{w,w^{\prime }}\in E^{\Lambda }$. The intervention indicator vector defined for position w is $I_{w}={\left(I_{e_{w,w^{\prime }}}\right)}_{w^{\prime }\in ch\left(w\right)}$. Define the *intervened position* to be the position whose floret F(w) is assigned a new probability distribution under an intervention. Let $w^{*}$ denote the set of intervened positions. Then, under a remedial intervention, $w^{*}\subseteq W^{\Delta }$. If $I_{e_{w,w^{\prime }}}=1$, then this means the root cause represented on $e_{w,w^{\prime }}$ is intervened and $w\in w^{*}$. We next formalize the manipulation of the probability distributions over $F(w^{*})$.

Definition 1 (Stochastic manipulations) A manipulation on a CEG C is called *stochastic* if there exists a set of positions $w^{*}\subseteq W$ such that

1. for each $w\in w^{*}$, there is a well‐defined map Γ updating the transition probabilities vector $\theta_{w}={\left(\theta_{w,w^{\prime }}\right)}_{w^{\prime }\in ch\left(w\right)}$
(6) $$\Gamma :\theta_{w}\mapsto {\hat{\theta }}_{w},$$
  where ${\hat{\theta }}_{w}={\left({\hat{\theta }}_{w,w^{\prime }}\right)}_{w^{\prime }\in ch\left(w\right)}$ denotes the postintervention transition probabilities vector;
2. the new transition probabilities vector ${\hat{\theta }}_{w}$ satisfies ${\hat{\theta }}_{w}\neq \theta_{w}$, $\sum_{w^{\prime }\in ch\left(w\right)}{\hat{\theta }}_{w,w^{\prime }}=1$ and ${\hat{\theta }}_{w,w^{\prime }}\in \left(0,1\right)$ for $w^{\prime }\in ch\left(w\right)$;
3. for a position $w\in W_{\Lambda (w^{*})}\setminus w^{*}$, that is, a position that lies on any of the paths passing through $w^{*}$ and is not an intervened position, the corresponding transition probabilities vector remains the same as the preintervention version: ${\hat{\theta }}_{w}=\theta_{w}$, here $\Lambda \left(w^{*}\right)=\cup_{w\in w^{*}}\Lambda \left(w\right)$ denote the *intervened paths*;
4. for a position $w^{\prime }\in ch\left(pa\left(w^{*}\right)\right)\setminus w^{*}$, that is, one that shares the same parent as $w^{*}$ but which is not an intervened position, ${\hat{\theta }}_{pa\left(w^{\prime }\right),w^{\prime }}=0$.

The simplest scenario for the manipulated transition probabilities ${\hat{\theta }}_{w^{*}}$ is when the values of ${\hat{\theta }}_{w^{*}}$ are known. But these values may not necessarily be available, in which case we have a more complex scenario where we are required to learn these values from an inferential framework.

We begin with the simplest scenario when ${\hat{\theta }}_{w^{*}}$ are known. In this case, the postintervention path related probabilities can be evaluated. Let π(·) and $\hat{\pi }\left(\cdot \right)$ denote the pre‐ and postintervention path‐related probability, respectively. Let $E\left(w^{*}\right)=\cup_{w\in W}E\left(w\right)$ denote the emanating edges of the intervened positions, $E_{\lambda }$ denote the set of edges traversed by the root‐to‐sink path λ. Then, for $\lambda \in \Lambda_{C}$,

(7) $$\hat{\pi }\left(\lambda \right)=\left\{\begin{array}{cc} \frac{\prod_{e\in E_{\lambda }}\theta_{e}}{\prod_{e^{\prime }\in E\left(w^{*}\right)\cap E_{\lambda }}\theta_{e^{\prime }}}\times \prod_{e^{\prime }\in E\left(w^{*}\right)\cap E_{\lambda }}{\hat{\theta }}_{e^{\prime }} & \text{if}\;\lambda \in \Lambda (w^{*}),\\ 0 & \text{otherwise.} \end{array}\right.$$

Given the intervened paths $\Lambda (w^{*})$, let ${\hat{C}}^{\Lambda (w^{*})}$ denote the topology of the conditioned CEG (Thwaites, 2013) constructed with respect to $\Lambda (w^{*})$. Let ${\hat{\pi }}^{\Lambda (w^{*})}\left(\cdot \right)$ denote the path related probability of ${\hat{C}}^{\Lambda (w^{*})}$, $W_{\lambda }$ denote the set of vertices traversed by λ. Then,

(8) $${\hat{\pi }}^{\Lambda (w^{*})}\left(\lambda \right)=\prod_{\begin{array}{c} w_{i},w_{j}\in W_{\lambda },\\ w_{i}=pa\left(w_{j}\right) \end{array}}{\hat{\pi }}^{\Lambda (w^{*})}\left(w_{j}|w_{i}\right)=\prod_{\begin{array}{c} w_{i},w_{j}\in W_{\lambda },\\ w_{i}=pa\left(w_{j}\right) \end{array}}\frac{\sum_{\lambda \in \Lambda (w^{*})}\hat{\pi }\left(\lambda ,\Lambda \left(e_{w_{i},w_{j}}\right)\right)}{\sum_{\lambda \in \Lambda (w^{*})}\hat{\pi }\left(\lambda ,\Lambda \left(w_{i}\right)\right)}.$$

Definition 2 (The manipulated CEG) The manipulated CEG of a remedial intervention with respect to $\Lambda (w^{*})$ has a topology $\hat{C}={\hat{C}}^{\Lambda (w^{*})}=\left(\hat{V},\hat{E}\right)$ and transition probabilities ${\hat{\theta }}^{*}$.

- The vertex set is $\hat{V}=W_{\Lambda (w^{*})}=\cup_{\lambda \in \Lambda (w^{*})}W_{\lambda }$;
- The edge set is $\hat{E}=E_{\Lambda (w^{*})}=\cup_{\lambda \in \Lambda (w^{*})}E_{\lambda }$;
- The transition probabilities are evaluated via Equation (8), Equation (7), and the specification in Definition 1.

We can now formalize the transformation of an idle CEG to a manipulated CEG under the remedial intervention. Let $\zeta_{w^{*}}$ denote the map indexed by the intervened positions $w^{*}$ corresponding to this transformation. Then,

(9) $$\zeta_{w^{*}}:\left(C,\theta \right)\mapsto \left(\hat{C},{\hat{\theta }}^{*}\right).$$

This map is well‐defined if and only if the map Γ that updates the transition probabilities, see Equation (6) in Definition 1, is well‐defined. Here, the intervened positions are fixed, while as discussed in Section 3.2, they might not be deterministic given the observations of remedial intervention and partial failure paths and the root causes needs to be inferred through the map $\rho :\left(A^{O},\lambda^{O}\right)\mapsto I_{E^{\Delta }}$. This map corresponds to Equation (5). Then, ζ∘Γ∘ρ allows us to construct the manipulated CEG.

**FIGURE 3:**
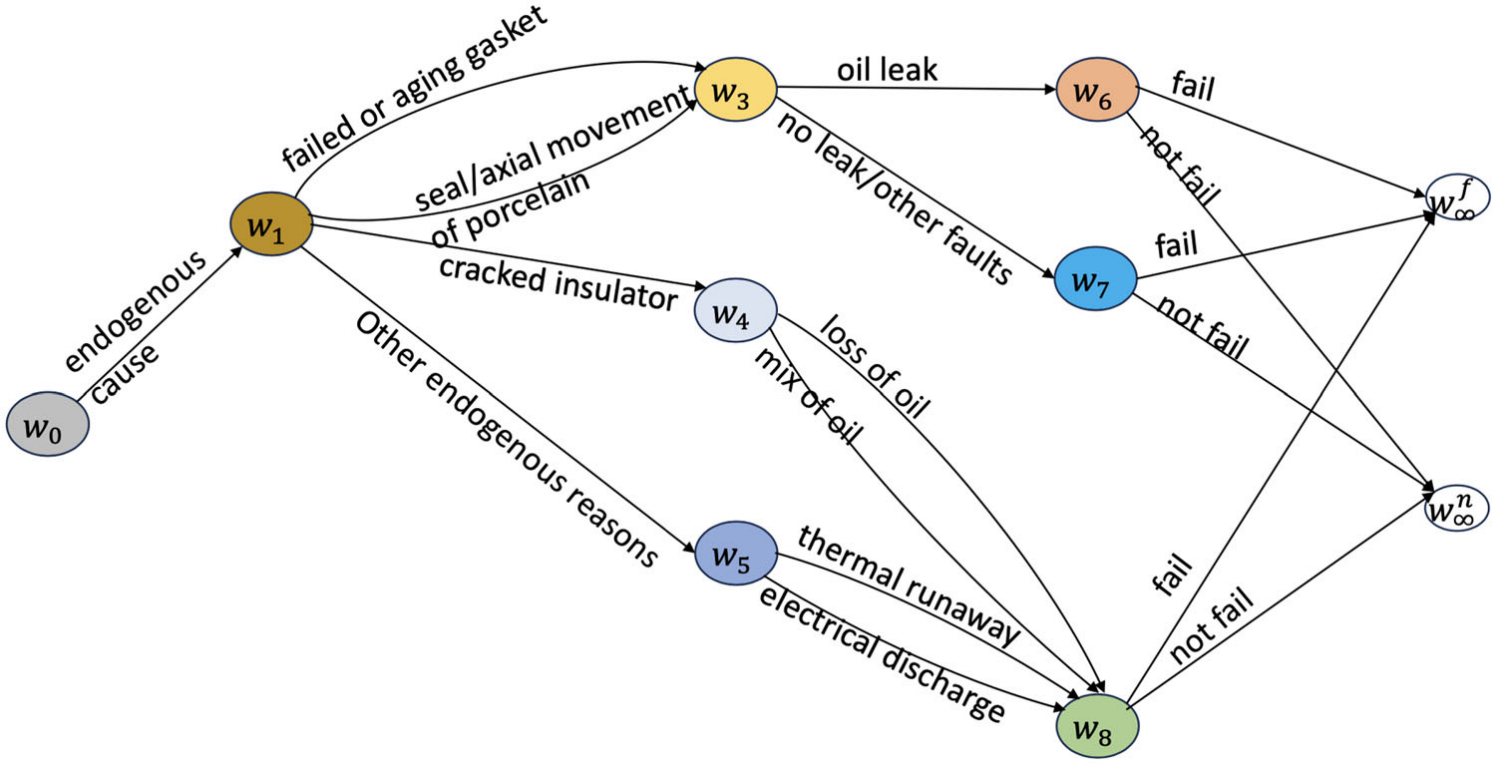
The manipulated chain event graph (CEG).

An example of a manipulated CEG is given in Figure 3. This corresponds to the idle CEG in Figure 1B for the bushing system and an imperfect remedial intervention that did not restore the status of the machine to AGAN. Suppose defects in gasket or porcelain lead to the failure. Then, the intervened position in $w^{*}=\left\{w_{1}\right\}$ and we can explore the effect of the remedial intervention by stochastically manipulating the distribution over $F(w_{1})$. The composition of stages may change when transforming from C to $\hat{C}$. The position $w_{8}$ in the idle system contains a single stage $u_{7}$. This stage consists of six situations that consist of situations $\left\{v_{7},v_{8},v_{13},v_{14},v_{15},v_{16}\right\}$. While in the manipulated CEG, vertices $v_{7},v_{8}$ are not traversed by any path in $\Lambda (v_{1})$ in the event tree, where $w_{1}=\left\{u_{1}\right\}=\left\{v_{1}\right\}$. The root floret $F(w_{0})$ is associated with the root cause classifier. Here, the manipulated CEG is conditioned on $\Lambda (w_{1})$, so ${\hat{\pi }}^{\Lambda (w_{1})}\left(w_{1}|w_{0}\right)=1$ and we only concern about the endogenous causes. In fact, exogenous root causes, such as lightening, are difficult to be remedied.

The manipulated CEG is associated with an intervened model and expresses what might happen had some variables been controlled under some hypothetical intervention. It allows us to identify the effect of some form of controls, for example, fixing a root cause, from the observed data and interpret it causally.

### 3.4 An inferential framework using the causal algebras

Of course, in practice, we need to estimate the parameters appearing in the formulas above before preceding to identify the causal effects which will be discussed in the next section. However, from a Bayesian perspective this is actually straightforward. Barclay et al. (2013) and Collazo et al. (2018) have established a conjugate analysis on the noncausal CEG which translates seamlessly into this new causal setting. Here, we only give an example of how Bayesian predictive inference can be performed. Let $f(\theta_{j,w}|\alpha_{w})$ denote the prior distribution of $\theta_{j,w}$, which is the transition probability vector of position w for individual j. Let $\chi_{j,e}$ denote whether d‐event on edge e is observed for individual j. The parameters of the prior is the vector $\alpha_{w}$. Let $\gamma_{a,\lambda }$ denote the parameter for the probability distribution of $I_{j,E^{\Delta }}|A_{j}^{O},\lambda_{j}^{O}$, with subscripts a denote the maintenance and λ denote the paths. Let $f(\gamma_{a,\lambda }|\beta )$ denote the prior of $\gamma_{a,\lambda }$ with parameter vector β. Then the posterior can be written as:

(10)

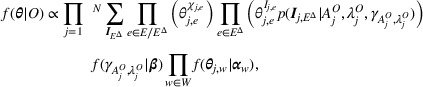

where O denotes the observations. We can sample $\gamma_{a,\lambda }$ from the posterior and simulate the root causes through simulating $I_{E^{\Delta }}$. Then, sampling θ from the posterior and further sampling the postintervened probabilities by the transformation $\Gamma ({\left\{\theta_{w}\right\}}_{w:I_{w^{\Delta }}=1})$. Then, we can find the predictive distribution over the paths by simulating the paths from the postintervened transition probabilities. Yu et al. (2020) and Yu and Smith (2021a) gave examples of implementing Bayesian inference with the customized causal algebras on CEGs for reliability analysis.

## 4 CAUSAL IDENTIFIABILITY OF A REMEDIAL INTERVENTION ON THE CEG

### 4.1 The expression of the causal query

For variable X represented on the causal BNs, the do‐operation (Pearl, 2009) that forces X to take value x is denoted by do(X=x). If we are interested in the effect of this intervention on another variable Y, then the causal query to be estimated is p(y|do(x)). This do‐operator corresponds to the singular manipulation on the CEG that forces $\Lambda_{x}$ to be traversed, where $\Lambda_{x}$ here is the collection of root‐to‐sink paths passing along the edges labeled by x. Analogously to p(y|do(x)), here we identify the effect of forcing x to happen on another event y through estimating the probability $\pi (\Lambda_{y}||\Lambda_{x})$. Here, the notation || plays a similar role as the do‐operator which imposes an intervention onto the tree (Thwaites, 2013). So, notationally $\pi (\Lambda_{y}\left|\right|\Lambda_{x})=\pi \left(\Lambda_{y}|do\left(\Lambda_{x}\right)\right)$.

We have explained that a remedial intervention imposes a stochastic manipulation on the probability distributions $\theta_{w^{*}}$ given the intervened positions $w^{*}$ and the postintervention transition probabilities assigned to $F(w^{*})$ is ${\hat{\theta }}_{w^{*}}$.Given ${\hat{\theta }}_{w^{*}}$, the causal query of a remedial intervention is

(11) $$\pi (\Lambda_{y}||{\hat{\theta }}_{w^{*}}).$$

Given an intervention $a^{O}$, we are interested in $\pi (\Lambda_{y}|do\left(a^{O}\right))$. However, $a^{O}$ is external to the system represented by the CEG. To identify this causal quantity, we transform the intervention onto the CEG using the formulation explained in the previous section. Recall that the intervened positions are identified through the map ρ, the transition probabilities are updated through the map Γ, and the manipulated CEG is then obtained through ξ. Thus, when the remedial intervention is perfect, that is, δ=1, the causal query can be expressed as

(12) $$\pi (\Lambda_{y}||\Gamma \left({\left\{\theta_{w}\right\}}_{w:I_{e_{w,w^{\prime }}}=1}\right)).$$

When the remedial intervention is imperfect or uncertain, that is, δ=0, we estimate the causal effect

(13) $$\begin{array}{ccc} \sum_{I_{E^{\Delta }}} & & \sum_{a^{u}\in A}\pi (\Lambda_{y}||\Gamma \left({\left\{\theta_{w}\right\}}_{w:I_{e_{w,w^{\prime }}}=1}\right))\\ & & p\left(I_{E^{\Delta }}|a^{O},a^{U},\lambda^{O},\delta =0\right)p\left(a^{U}|a^{O},\lambda^{O},\delta =0\right), \end{array}$$

where the postintervention transition probabilities ${\hat{\theta }}_{w^{*}}$ are obtained from observations via Γ∘ρ. For either type of remedial intervention, we need to identify $\pi (\Lambda_{y}||\Gamma \left({\left\{\theta_{w}\right\}}_{w:I_{e_{w,w^{\prime }}}=1}\right))$. In this section, we only focus on the quantity $\pi (\Lambda_{y}||{\hat{\theta }}_{w^{*}}))$ with a known ${\hat{\theta }}_{w^{*}}=\Gamma \left({\left\{\theta_{w}\right\}}_{w:I_{e_{w,w^{\prime }}}=1}\right)$. Note that given the idle CEG we can construct the manipulated CEG with ${\hat{\theta }}^{*}$. Based on this knowledge, we next show the effect of the stochastic manipulation $\pi (\Lambda_{y}||{\hat{\theta }}_{w^{*}}))$ is identifiable given the idle CEG and the observable information.

### 4.2 Causal effect identifiability of stochastic manipulations

A *fine cut* (Wilkerson, 2020) is defined to be a set of vertices $W^{\prime }\subset W_{T}$ so that $\cup_{w\in W^{\prime }}\Lambda \left(w\right)=\Lambda_{C}$. The intervened positions under a remedial intervention are not necessarily a fine cut. If $w^{*}$ is a fine cut of C, then the intervened paths are the set of all root‐to‐sink paths on the CEG, that is, $\Lambda \left(w^{*}\right)=\Lambda_{C}$. When the manipulations are asymmetric or the processes modeled on the idle CEG are asymmetric, $w^{*}$ might not be a fine cut. A CEG conditional on the intervened paths $\Lambda (w^{*})$ can be constructed. Such a *conditioned CEG* has structure $C^{\Lambda (w^{*})}=\left(V^{*},E^{*}\right)$, where $V^{*}=W_{\Lambda (w^{*})}\cup w_{\infty }^{f}\cup w_{\infty }^{n}$ and $E^{*}=E_{\Lambda (w^{*})}$. The transition probabilities are $\theta^{*}={\left\{\theta_{w}^{*}\right\}}_{w\in W_{\Lambda (w^{*})}}$, where $\theta_{w,w^{\prime }}^{*}=\pi^{\Lambda (w^{*})}\left(w_{j}|w_{i}\right)$ which is evaluated as:

(14) $$\pi^{\Lambda (w^{*})}\left(w_{j}|w_{i}\right)=\frac{\sum_{\lambda \in \Lambda (w^{*})}\pi \left(\lambda ,\Lambda \left(e_{w_{i},w_{j}}\right)\right)}{\sum_{\lambda \in \Lambda (w^{*})}\pi \left(\lambda ,\Lambda \left(w_{i}\right)\right)}.$$

So the conditioned CEG differs from the manipulated CEG in that it inherits preintervention conditional probabilities.

Analogously to the singular intervention (Thwaites et al., 2010; Thwaites, 2013), to identify the causal effects of a stochastic manipulation on $\theta_{w^{*}}$, we need to estimate the probability $\pi (\Lambda_{y}||{\hat{\theta }}_{w^{*}})$ from the conditioned CEG $\left(C^{\Lambda (w^{*})},\theta^{*}\right)$.

Let the intervened d‐events be $x\left(E\left(w^{*}\right)\right)=\cup_{w\in w^{*}}\cup_{e\in E(w)}x\left(e\right)$, which are labels of the edges emanating from the intervened positions. For remedial interventions, they are a subset of root causes.

Given $x(E\left(w^{*}\right))$ and ${\hat{\theta }}_{w^{*}}$, estimating $\pi (\Lambda_{y}||{\hat{\theta }}_{w^{*}})$ from $\left(C^{\Lambda (w^{*})},\theta^{*}\right)$ is equivalent to manipulating each d‐event $x\in x(E\left(w^{*}\right))$ with probability $\pi (\Lambda_{x}||{\hat{\theta }}_{w^{*}})$. Note that estimating the causal query in this way is standard for causal algebras. For example, Pearl (2009) has suggested estimating the causal effects of a stochastic policy from the an unmanipulated causal BN in a similar way. Following this idea, we can formulate the causal query as follows.

Definition 3 (Causal effect of a stochastic manipulation) Given the intervened d‐events $x(E\left(w^{*}\right))$ and the conditioned CEG $\left(C^{\Lambda (w^{*})},\theta^{*}\right)$, the causal effect of the stochastic manipulation of ${\hat{\theta }}_{w^{*}}$ on the d‐event y is a function from the stochastically manipulated probability vectors ${\hat{\theta }}_{w^{*}}$ to the space of path‐related probability distributions on $\Lambda_{y}$ which can be expressed as:

(15) $$\pi (\Lambda_{y}\left|\right|{\hat{\theta }}_{w^{*}})=\sum_{x\in x(E\left(w^{*}\right))}\pi^{\Lambda (w^{*})}(\Lambda_{y}\left|\right|\Lambda_{x}){\hat{\pi }}^{\Lambda (w^{*})}\left(\Lambda_{x}\right),$$

where

(16) $$\pi (\Lambda_{y}\left|\right|\Lambda_{x},{\hat{\theta }}_{w^{*}})=\pi^{\Lambda (w^{*})}(\Lambda_{y}\left|\right|\Lambda_{x}),$$

(17) $$\pi (\Lambda_{x}\left|\right|{\hat{\theta }}_{w^{*}})={\hat{\pi }}^{\Lambda (w^{*})}\left(\Lambda_{x}\right).$$

Given this definition, the causal effects of such a stochastic manipulation is identifiable if and only if $\pi^{\Lambda (w^{*})}(\Lambda_{y}\left|\right|\Lambda_{x})$ can be uniquely estimated for every $x\in x(E\left(w^{*}\right))$ given the CEG and the observations. Recall that the intervened positions $w^{*}$ may not form a fine cut. So, $\sum_{x\in x(E\left(w^{*}\right))}\pi \left(\Lambda_{x}\right)$ may not be equal to 1 unless conditional on $\Lambda (w^{*})$. But when the intervened positions $w^{*}$ form a fine cut, then $\Lambda \left(w^{*}\right)=\Lambda_{C}$ and $\sum_{x\in x(E\left(w^{*}\right))}\pi \left(\Lambda_{x}\right)=1$.

Note that $\pi^{\Lambda (w^{*})}(\Lambda_{y}\left|\right|\Lambda_{x})$ is estimated from the conditioned idle CEG $\left(C^{\Lambda (w^{*})},\theta^{*}\right)$. So, the path‐related probability is π(·) instead of $\hat{\pi }\left(\cdot \right)$. On the other hand, ${\hat{\pi }}^{\Lambda (w^{*})}\left(\Lambda_{x}\right)$ is assumed to be known given ${\hat{\theta }}_{w^{*}}$. This is the postintervention probability of a unit passing along the paths $\Lambda_{x}$ a stochastic manipulation on ${\hat{\theta }}_{w^{*}}$, where $\Lambda_{x}\subseteq \Lambda \left(w^{*}\right)$. The postintervention path related probabilities ${\hat{\pi }}^{\Lambda (w^{*})}\left(\Lambda_{x}\right)$ can be derived using Equations (7) and (8).

No restriction is imposed on the intervened d‐events. Under remedial interventions, however, these are normally root causes. Yu and Smith (2021b) also discussed another type of intervention in reliability in which case the intervened d‐events can be a component, a typical symptom, and so on, depending on the maintenance scheduled and conducted by the engineers. Definition 3 generically formulates a causal query from a stochastic manipulation which should not be restricted for remedial interventions.

Next we show the causal identifiability of the stochastic manipulation.

Proposition 1 Suppose a remedial intervention imposes a stochastic manipulation on the distributions of $F(w^{*})$. Then given the postintervention transition probabilities ${\hat{\theta }}_{w^{*}}$, the effects of this intervention are identifiable if and only if $\pi^{\Lambda (w^{*})}(\Lambda_{y}\left|\right|\Lambda_{x})$ can be uniquely estimated for every $x\in x(E\left(w^{*}\right))$ given the CEG and the observations.

(1) $\pi^{\Lambda (w^{*})}(\Lambda_{y}\left|\right|\Lambda_{x})\;\text{is identifiable}\Rightarrow \pi (\Lambda_{y}\left|\right|{\hat{\theta }}_{w^{*}})\;\text{is identifiable}$: from Equation (15), it is straightforward to see that if $\pi^{\Lambda (w^{*})}(\Lambda_{y}\left|\right|\Lambda_{x})$ is identifiable for all $x\in x(E\left(w^{*}\right))$, then $\pi (\Lambda_{y}||{\hat{\theta }}_{w^{*}})$ can be estimated, since ${\hat{\pi }}^{\Lambda (w^{*})}\left(\Lambda_{x}\right)$ is determined externally.

(2) $\pi (\Lambda_{y}\left|\right|{\hat{\theta }}_{w^{*}})\;\text{is identifiable}\Rightarrow \pi^{\Lambda (w^{*})}(\Lambda_{y}\left|\right|\Lambda_{x})\;\text{is identifiable}$: if there exists a d‐event $x\in x(E\left(w^{*}\right))$ so that $\pi^{\Lambda (w^{*})}(\Lambda_{y}\left|\right|\Lambda_{x})$ cannot be estimated, then the effects of the manipulation of ${\hat{\theta }}_{w,w(x)}$ are not identifiable. Since $e_{w,w(x)}\in E\left(w^{*}\right)$ and $w\in w^{*}$, ${\hat{\theta }}_{w,w(x)}\in {\hat{\theta }}_{w^{*}}$. Therefore, the effects of ${\hat{\theta }}_{w^{*}}$ could not be fully estimated, so $\pi (\Lambda_{y}||{\hat{\theta }}_{w^{*}})$ could not be estimated, giving the required contradiction. So the identifiability of $\pi (\Lambda_{y}||{\hat{\theta }}_{w^{*}})$ implies the identifiability of $\pi^{\Lambda (w^{*})}(\Lambda_{y}\left|\right|\Lambda_{x})$.□

Therefore, the identifiability of $\pi^{\Lambda (w^{*})}(\Lambda_{y}\left|\right|\Lambda_{x})$ is a necessary and sufficient condition for the identifiability of $\pi (\Lambda_{y}||{\hat{\theta }}_{w^{*}})$. Thwaites (2013) has proved the identifiability of a singular manipulation on the CEG through adapting the backdoor and the front‐door theorems which are graphical tests designed by Pearl (2009) to examine the identifiability of the causal effects of an atomic intervention on the causal BN. For simplicity, here we only focus on extending the backdoor theorem here to prove causal identifiability. The idea is to find a partition of the root‐to‐sink paths $\Lambda_{C}$, denoted by $\Lambda_{z}$ so that the following criteria are satisfied.

Theorem 1 For any $e_{w,w^{\prime }}\in e\left(x\right)$, if

(18) $$\pi \left(\Lambda_{z}|\Lambda \left(w\right)\right)=\pi \left(\Lambda_{z}|\Lambda \left(e_{w,w^{\prime }}\right)\right)$$

(19) $$\pi \left(\Lambda_{y}|\Lambda_{x},\Lambda_{z}\right)=\pi \left(\Lambda_{y}|\Lambda \left(w\right),\Lambda_{x},\Lambda_{z}\right)=\pi \left(\Lambda_{y}|\Lambda \left(e_{w,w^{\prime }}\right),\Lambda_{z}\right)$$

hold for every element of $\left\{\Lambda_{z}\right\}$, then $\left\{\Lambda_{z}\right\}$ is the *backdoor partition* (Thwaites, 2013).

For a stochastic manipulation, as we have explained, we wish to estimate $\pi^{\Lambda (w^{*})}(\Lambda_{y}\left|\right|\Lambda_{x})$ from $\left(C^{\Lambda (w^{*})},\theta^{*}\right)$. So we can simply adapt the above two criteria as follows.

Theorem 2 For any $w\in w^{*}$, for all $x\in x(E\left(w^{*}\right))$ and any $e_{w,w^{\prime }}\in e\left(x\right)$ if

(20) $$\pi^{\Lambda (w^{*})}\left(\Lambda_{z}|\Lambda \left(w\right)\right)=\pi^{\Lambda (w^{*})}\left(\Lambda_{z}|\Lambda \left(e_{w,w^{\prime }}\right)\right)$$

and

(21) $$\pi^{\Lambda (w^{*})}\left(\Lambda_{y}|\Lambda \left(w\right),\Lambda_{x},\Lambda_{z}\right)=\pi^{\Lambda (w^{*})}\left(\Lambda_{y}|\Lambda \left(e_{w,w^{\prime }}\right),\Lambda_{z}\right)$$

hold for every element of $\left\{\Lambda_{z}\right\}$, then $\left\{\Lambda_{z}\right\}$ is the backdoor partition for identifying the effects of a remedial intervention.

Note that $w^{*}$ form a fine cut of $\Lambda (w^{*})$ and $ch(w^{*})$ also form a fine cut of $\Lambda (w^{*})$. The set of paths $\left\{\Lambda_{z}\right\}$ is a partition of $\Lambda (w^{*})$. Next we formalize the backdoor theorem for identifying the causal effects of a remedial intervention.

Theorem 3 The effects of a stochastic manipulation are identifiable whenever a backdoor partition $\left\{\Lambda_{z}\right\}$ can be found so that

(22) $$\pi (\Lambda_{y}\left|\right|{\hat{\theta }}_{w^{*}})=\sum_{x\in x(E\left(w^{*}\right))}\sum_{z}\pi^{\Lambda (w^{*})}\left(\Lambda_{y}|\Lambda_{x},\Lambda_{z}\right)\pi^{\Lambda (w^{*})}\left(\Lambda_{z}\right){\hat{\pi }}^{\Lambda (w^{*})}\left(\Lambda_{x}\right).$$

This holds when

(23) $$\pi^{\Lambda (w^{*})}\left(\Lambda_{y}|{\hat{\Lambda }}_{x},\Lambda_{z}\right)=\pi^{\Lambda (w^{*})}\left(\Lambda_{y}|\Lambda_{x},\Lambda_{z}\right),$$

here ${\hat{\Lambda }}_{x}$ denotes that there is a singular intervention on $\Lambda_{x}$ only, not on $\Lambda_{z}$, and

(24) $$\pi^{\Lambda (w^{*})}(\Lambda_{z}\left|\right|\Lambda_{x})=\pi^{\Lambda (w^{*})}\left(\Lambda_{z}\right).$$

It is now straightforward to deduce this from the two criteria in Theorem 2 and adapting the proof of the backdoor theorem for the singular intervention (Thwaites, 2013). We prove this theorem in the supplementary material. We allow flexibility in choosing z, where z can be a set of d‐events $z=\{z_{1},\text{…},z_{l}\}$. Then, $\Lambda_{z}=\left\{\Lambda_{z_{1}},\text{…},\Lambda_{z_{l}}\right\}$. We can also define z to be a set of stages, positions, or edges. Next, we give an example to show how to find an appropriate backdoor partition for a remedial intervention.

We can now illustrate how the formulas work for the bushing example.

Example 1 Recall that we let the intervened position be $w_{1}$ and the manipulated CEG is Figure 3. The intervened paths are $\Lambda_{x(e_{w_{1},w_{3}}^{1})}\cup \Lambda_{x(e_{w_{1},w_{3}}^{2})}\cup \Lambda_{x(e_{w_{1},w_{4}})}\cup \Lambda_{x(e_{w_{1},w_{5}})}$ 2. Suppose we are interested in how the maintenance will affect system failure. Then, $\Lambda_{y}=\Lambda_{\text{fail}}$.

Let $\Lambda_{z}=\left\{\Lambda_{z_{1}},\Lambda_{z_{2}}\right\}$ be a partition of $\Lambda (w_{1})$. We can define this partition according to the symptoms. Let $z_{1}$ be a set of d‐events: { oil leak, loss of oil, thermal runaway }, *that is*, $e\left(z_{1}\right)=\left\{e_{w_{3},w_{6}},e_{w_{4},w_{8}}^{1},e_{w_{5},w_{8}}^{1}\right\}$. Let $z_{2}$ be a set of d‐events: { no oil leak and no other faults, mix of oil, electrical discharge }. Then, $e\left(z_{2}\right)=\left\{e_{w_{3},w_{7}},e_{w_{4},w_{8}}^{2},e_{w_{5},w_{8}}^{2}\right\}$. Thus, $\Lambda_{z_{1}}=\Lambda_{x(e_{w_{3},w_{6}})}\cup \Lambda_{x(e_{w_{4},w_{8}}^{1})}\cup \Lambda_{x(e_{w_{5},w_{8}}^{1})}$, $\Lambda_{z_{2}}=\Lambda_{x(e_{w_{3},w_{7}})}\cup \Lambda_{x(e_{w_{4},w_{8}}^{2})}\cup \Lambda_{x(e_{w_{5},w_{8}}^{2})}$.

Suppose the postintervention probabilities ${\hat{\theta }}_{w_{1}}$ are known. Having a stochastic manipulation on the distribution over $F(w_{1})$ can be treated as having a singular manipulation on $e_{w_{1},w^{\prime }}$ with probability ${\hat{\theta }}_{w_{1},w^{\prime }}$, where $w^{\prime }\in ch\left(w_{1}\right)$. We can validate Theorem 2 for all $x\in x(E\left(w_{1}\right))$. To check the first criterion,

(25) $$\begin{array}{cc} \pi^{\Lambda (w_{1})}\left(\Lambda \left(e\left(z_{1}\right)\right)|\Lambda_{x(e_{w_{1},w_{3}}^{1})}\right) & =\frac{\theta_{w_{0},w_{1}}^{*}\theta_{w_{1},w_{3}}^{*1}\theta_{w_{3},w_{6}}^{*}}{\theta_{w_{0},w_{1}}^{*}\theta_{w_{1},w_{3}}^{*1}}=\theta_{w_{3},w_{6}}^{*}. \end{array}$$

We can also compute:

(26) $$\begin{array}{cc} & \pi^{\Lambda (w_{1})}\left(\Lambda \left(e\left(z_{1}\right)\right)|\Lambda \left(w_{1}\right)\right)\\ & =\frac{\theta_{w_{0},w_{1}}^{*}\left(\theta_{w_{1},w_{3}}^{*1}+\theta_{w_{1},w_{3}}^{*2}\right)\theta_{w_{3},w_{6}}^{*}+\theta_{w_{0},w_{1}}^{*}\theta_{w_{1},w_{4}}^{*}\theta_{w_{4},w_{8}}^{*1}+\theta_{w_{0},w_{1}}^{*}\theta_{w_{1},w_{5}}^{*}\theta_{w_{5},w_{8}}^{*1}}{\theta_{w_{0},w_{1}}^{*}}\\ & =\left(\theta_{w_{1},w_{3}}^{*1}+\theta_{w_{1},w_{3}}^{*2}\right)\theta_{w_{3},w_{6}}^{*}+\theta_{w_{1},w_{4}}^{*}\theta_{w_{4},w_{8}}^{*1}+\theta_{w_{1},w_{5}}^{*}\theta_{w_{5},w_{8}}^{*1}=\theta_{w_{3},w_{6}}^{*}. \end{array}$$

The last equality is valid because $e_{w_{3},w_{6}},e_{w_{4},w_{8}}^{1},e_{w_{5},w_{8}}^{1}$ have the same transition probabilities and $\theta_{w_{1},w_{3}}^{1*}+\theta_{w_{1},w_{3}}^{2*}+\theta_{w_{1},w_{4}}^{*}+\theta_{w_{1},w_{5}}^{*}=1$. So,

(27) $$\pi^{\Lambda (w_{1})}\left(\Lambda \left(e\left(z_{1}\right)\right)|\Lambda \left(e_{w_{1},w_{3}}^{1}\right)\right)=\pi^{\Lambda (w_{1})}\left(\Lambda \left(e\left(z_{1}\right)\right)|\Lambda \left(w_{1}\right)\right).$$

Following in this way, it is easy to check that the first criterion is satisfied for all manipulated x and partition z.

Now we check whether the second criterion is satisfied.

(28) $$\begin{array}{cc} \pi^{\Lambda (w_{1})}\left(\Lambda_{\text{fail}}|\Lambda \left(e\left(z_{1}\right)\right),\Lambda_{x(e_{w_{1},w_{3}}^{1})}\right) & =\pi^{\Lambda (w_{1})}\left(\Lambda_{\text{fail}}|\Lambda \left(e\left(z_{1}\right)\right)\cap \Lambda_{x(e_{w_{1},w_{3}}^{1})}\right)\\ & =\pi^{\Lambda (w_{1})}\left(\Lambda_{\text{fail}}|\Lambda \left(e_{w_{3},w_{6}}\right),\Lambda \left(e_{w_{1},w_{3}}^{1}\right)\right)\\ & =\pi^{\Lambda (w_{1})}\left(\Lambda_{\text{fail}}|\Lambda \left(e\left(z_{1}\right)\right),\Lambda \left(e_{w_{1},w_{3}}^{1}\right)\right). \end{array}$$

Using the same method, it is straightforward to check the second criterion is satisfied for $e\left(z_{1}\right),e\left(z_{2}\right)$ and all the intervened events. Therefore, $\Lambda_{z}$ is a backdoor partition.

As mentioned in the previous section, there is a special case of the stochastic manipulation that $w^{*}$ form a fine cut of the idle CEG. In this case,

(29) $$\pi (\Lambda_{y}\left|\right|{\hat{\theta }}_{w^{*}})=\sum_{x\in x(E\left(w^{*}\right))}\sum_{z}\pi \left(\Lambda_{y}|\Lambda_{x},\Lambda_{z}\right)\pi \left(\Lambda_{z}\right){\hat{\pi }}^{\Lambda (w^{*})}\left(\Lambda_{x}\right).$$

Then to identify the causal effects, we only need to show that for every $x\in x(E\left(w^{*}\right))$ we can find a backdoor partition satisfying the criteria in Theorem 1.

The formulas given in this section is useful because we can formally identify the efficacy of the intervention which is expressed through a quantitative probability score which is a function of terms we can identify from the idle system.

**FIGURE 4:**
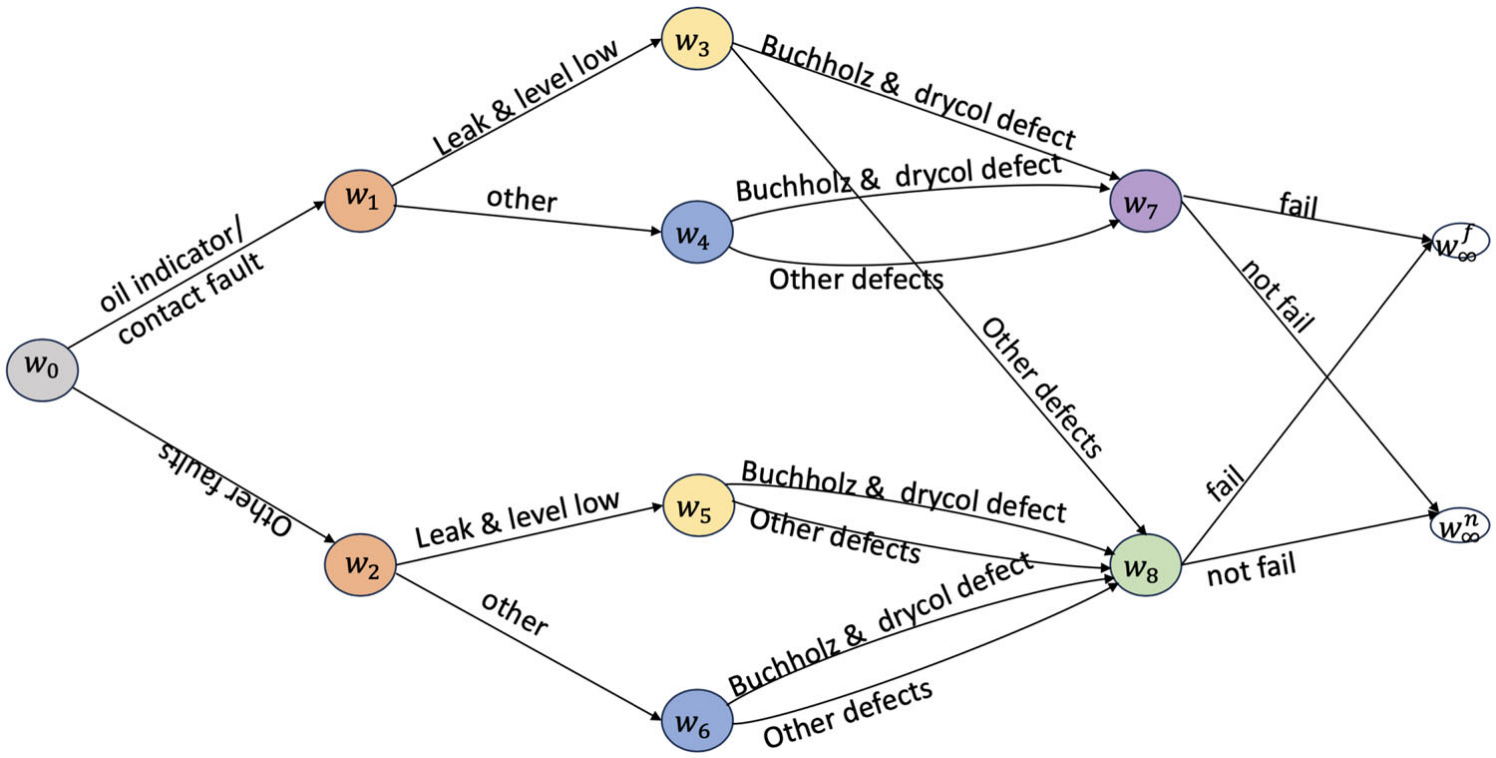
A hypothesized chain event graph (CEG) of a conservator system for the example in Section 4.2.

Example 2 Here, we give an example of a conservator system of a transformer (see Figure 4). The initial events are root causes: { oil indicator/contact fault, other fault }. Following the root causes, we attach the oil status of the transformer: { leak & level low, other }, where other refers to the condition when there is no leak and oil level is normal, or there is only oil leak, or only oil level is low. Defects in two components buchholz and drycol may occur after oil problems. Here we consider whether the two components are both faulty or otherwise—either is faulty or both are functioning. Then we attach the failure indicator.

Suppose there is a remedial intervention which replaced the deteriorated seal. This maintenance remedied the contact fault. In response to the intervention, the conditional probability assigned to $e_{w_{0},w_{1}}$ is expected to decrease and the probability distribution over $F(w_{0})$ is manipulated.

The intervened paths are $\Lambda \left(w_{0}\right)=\Lambda_{C}$. So the manipulated CEG has the same topology as the idle CEG. Let the effect event be system failure, that is, $\Lambda_{y}=\Lambda_{\text{fail}}$. We estimate the effect through:

(30) $$\pi (\Lambda_{\text{fail}}\left|\right|{\hat{\theta }}_{w_{0}})=\pi (\Lambda_{x_{f,1}}||\Lambda \left(e_{w_{0},w_{1}}\right)){\hat{\theta }}_{w_{0},w_{1}}+\pi (\Lambda_{x_{f,1}}||\Lambda \left(e_{w_{0},w_{2}}\right)){\hat{\theta }}_{w_{0},w_{2}}.$$

We can define the backdoor partition in terms of stages. The buchholz and drycol status is represented by the florets: $F\left(w_{3}\right),F\left(w_{4}\right),F\left(w_{5}\right),F\left(w_{6}\right)$. Note that $w_{3}$ and $w_{5}$ are in the same stage, while $w_{4}$ and $w_{6}$ are in the same stage. These stages lie upstream of e(fail) and downstream of the intervened root causes. Let $\Lambda_{z_{1}}=\left\{\Lambda \left(w_{3}\right),\Lambda \left(w_{5}\right)\right\}$ and $\Lambda_{z_{2}}=\left\{\Lambda \left(w_{4}\right),\Lambda \left(w_{6}\right)\right\}$. Then, $\Lambda_{z}=\left\{\Lambda_{z_{1}},\Lambda_{z_{2}}\right\}$ partitions $\Lambda_{C}$ and each $z_{i}$ is associated with a single stage.

We check the first criterion by showing for any $z_{i}$,

(31) $$\pi \left(\Lambda_{z_{i}}|w_{0}\right)=\pi \left(\Lambda_{z_{i}}\right).$$

For $z_{1}$,

(32) $$\pi \left(\Lambda_{z_{1}}\right)=\theta_{w_{0},w_{1}}\theta_{w_{1},w_{3}}+\theta_{w_{0},w_{2}}\theta_{w_{2},w_{5}}=\theta_{w_{1},w_{3}}=\theta_{w_{2},w_{5}}.$$

Given a singular intervention on $e_{w_{0},w_{1}},\theta_{w_{1},w_{3}}=\pi \left(\Lambda_{z_{1}}|\Lambda \left(e_{w_{0},w_{1}}\right)\right)$, and $\theta_{w_{2},w_{5}}=\pi \left(\Lambda_{z_{1}}|\Lambda \left(e_{w_{0},w_{2}}\right)\right)$. Similarly, it is easy to show that $\pi \left(\Lambda_{z_{2}}|\Lambda \left(e_{w_{0},w_{1}}\right)\right)=\pi \left(\Lambda_{z_{2}}\right)$ and $\pi \left(\Lambda_{z_{2}}|\Lambda \left(e_{w_{0},w_{2}}\right)\right)=\pi \left(\Lambda_{z_{2}}\right)$. So the first criterion is satisfied.

When there is a singular intervention on $e_{w_{0},w_{1}}$,

(33) $$\begin{array}{ccc} \pi (\Lambda_{\text{fail}}|\Lambda \left(w_{0}\right),\Lambda_{x},\Lambda_{z_{1}})) & & =\pi (\Lambda_{\text{fail}}|\Lambda \left(w_{0}\right),\Lambda \left(e_{w_{0},w_{1}}\right),\Lambda_{z_{1}}))\\ & & =\pi (\Lambda_{\text{fail}}|\Lambda \left(e_{w_{0},w_{1}}\right),\Lambda_{z_{1}})), \end{array}$$

and

(34) $$\begin{array}{ccc} \pi (\Lambda_{\text{fail}}|\Lambda \left(w_{0}\right),\Lambda_{x},\Lambda_{z_{2}})) & & =\pi (\Lambda_{\text{fail}}|\Lambda \left(w_{0}\right),\Lambda \left(e_{w_{0},w_{1}}\right),\Lambda_{z_{1}}))\\ & & =\pi (\Lambda_{\text{fail}}|\Lambda \left(e_{w_{0},w_{1}}\right),\Lambda_{z_{2}})). \end{array}$$

Similarly, when there is a singular intervention on $e_{w_{0},w_{2}}$, we reach the same expressions as above with $e_{w_{0},w_{1}}$ replaced by $e_{w_{0},w_{2}}$. So the second criterion in Theorem 2 is satisfied. Therefore, $\left\{\Lambda_{z_{1}},\Lambda_{z_{2}}\right\}$ forms a backdoor partition here.

## 5 A SIMULATION STUDY

**FIGURE 5:**
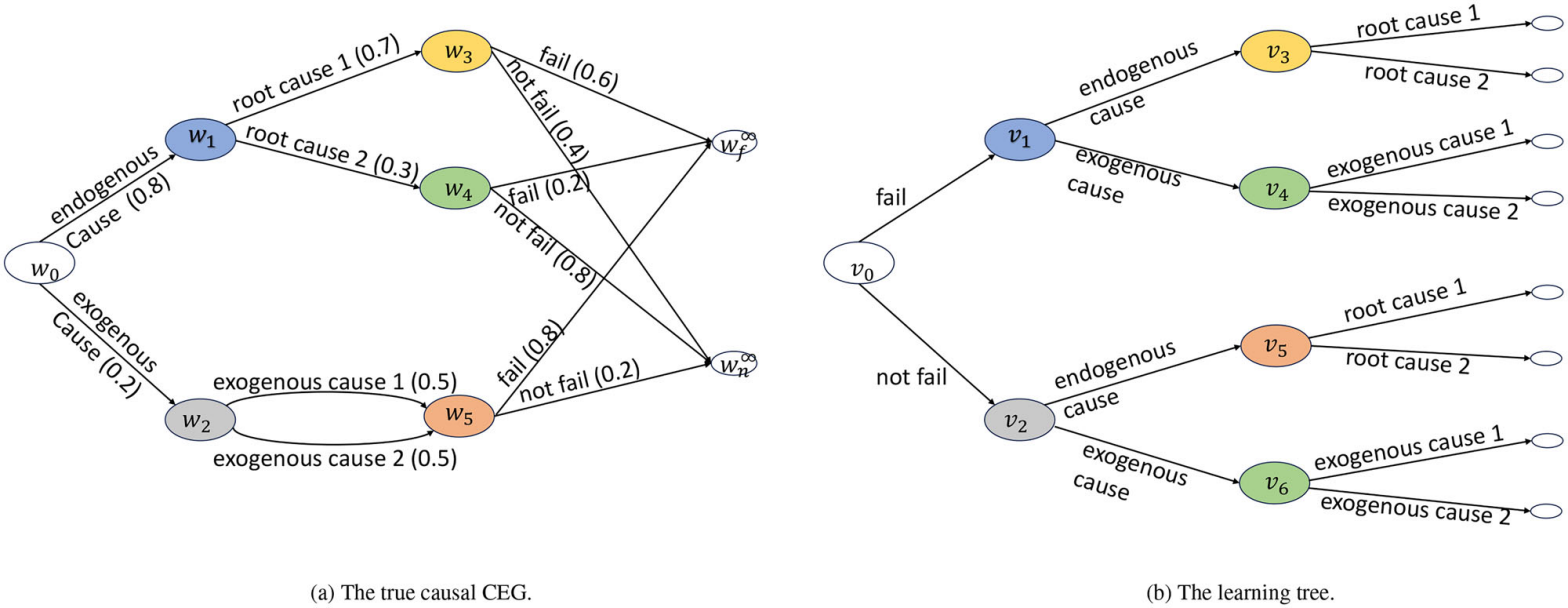
Trees for the simulation study. Numbers in brackets are the true parameters. We use the same color palette for the trees. Vertices in Panels (A) and (B) with the same color do not share the same transition probability vector.

The engineer reports in the domain we studied are sensitive. So instead we demonstrate how to apply the causal algebras proposed in previous sections in practice on a simulated data set.^3^ Let Figure 5A be the ground truth causal CEG^4^ and we simulate a synthetic data set comprising 5000 cases. There are 2698 failure cases which emulate the information extracted from the failure report. The rest emulate the record of preventive maintenance (Yu & Smith, 2021a). We generate perfect remedies by simply assuming

(35) $$\begin{array}{cc} p & \left(\delta =0|\text{endogenous cause},\text{fail},\text{root cause 1}\right)\\ & =p(\delta =0|\text{endogenous cause},\text{fail},\text{root cause 2}) \end{array}$$

(36) $$\begin{array}{cc} & =p(\delta =0|\text{endogenous cause},\text{fail})=0.3. \end{array}$$

For the cases with nonperfect remedy, we assume the failure processes are only partially observed.

In practice, the explanations of the effects of remedial interventions will have been extracted from engineer's reports either manually or automatically (Yu, 2021). The tree consistent with these explanations and data should begin with the failure indicator. Thus, we transform Figure 5A to an equivalent tree in Figure 5B, where the transition probabilities can be calculated using Bayes rule. We call it the *learning tree*.

Suppose we know the ground truth event tree for this system, which shares the same the topology as the learning tree. We next learn the parameter and stages of the tree from the simulated data. The parameters can be learned in a Bayesian framework based on Equation (10) using the Metropolis–Hastings algorithm. Here, we simply assume Dirichlet priors for transition probability vectors, where $\theta_{w}∼\text{Dirichlet}\left(\alpha_{w}\right)$. The choice of $\alpha_{w}$ is discussed in the supplementary material. The structure of the CEG (i.e., stages) is learned using the maximum a posterior (MAP) algorithm—this is the most used Bayesian structural learning method for CEGs (see Barclay et al., 2013; Cowell & Smith, 2014; Strong, 2023; Yu et al., 2020; Yu & Smith, 2021a). After learning the best‐scored CEG on the learning tree, we transform it back to the causal CEG. This allows us to perform the causal analysis described in Section 4. To impose the effects of remedies, we manipulate $\theta_{w_{1}}$ via Γ. Here, we simply let $\frac{{\hat{\theta }}_{w_{1},w_{3}}}{{\hat{\theta }}_{w_{1},w_{4}}}=\frac{\theta_{w_{1},w_{3}}}{\theta_{w_{1},w_{4}}}\times 0.8^{\frac{N_{w_{1},w_{3}}-N_{w_{1},w_{4}}}{N}}$, where $N_{w_{i},w_{j}}$ counts the observations associated with $e_{w_{i},w_{j}}$. Other choices of Γ have been discussed by Yu et al. (2020) and Yu (2021).

**FIGURE 6:**
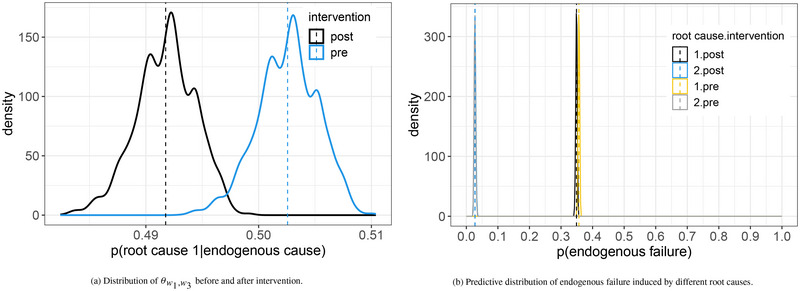
Results of simulation study.

The best‐scored tree learned from the synthetic data has the same structure as Figure 5B where $v_{3}$ and $v_{5}$ are in different stages while $v_{4}$ and $v_{8}$ are in the same stage. Its MAP score is −7212.494, 2698.319 higher than the score of the tree with $v_{3}$ and $v_{5}$ in the same stage. The sensitivity analysis is summarized in Section 2 of the supplementary material. The manipulation shifts the distribution of $\theta_{w_{1},w_{3}}$ to the left, as shown in Figure 6A. The posterior mean of $\theta_{w_{1},w_{3}}$ is reduced to 0.492 from 0.503 after manipulation. We can then predict the system failure induced by the two root causes based on the estimated distribution of ${\hat{\theta }}_{w_{1}}$ (see Figure 6).

## 6 DISCUSSION

In this article, we have shown the flexibility of the semantics of a CEG in representing asymmetric processes and capturing the effects of various interventions even when the manipulations are asymmetric. Given a context‐specific CEG for a particular system, we can design the bespoke causal algebras for different types of remedial interventions. We can predict a machine's failure probability by imposing the underlying stochastic manipulations to the idle system so that we can identify the effects of the remedial intervention through finding appropriate backdoor partition on the CEG. The graphical methodology we have described here therefore provides an excellent framework for translating established formal causal analyses so that these can be embedded into mainstream system reliability.

The original domain that motivated this formal development did not contain the case where the discovery of a fault would encourage an improvement of the system. However, a referee pointed out there are many domains where a fault would provoke a system upgrade.^5^ In such cases, the failure rate could be reduced over the entire lifetime after maintenance. Thus, more complex manipulations, beyond restoring root causes to AGAN, should be considered. Semi‐Markov processes can be modeled on dynamic CEGs (Barclay et al., 2015), enabling us to generalize our proposed algebras and manipulate failure rates directly. Our previous work (Yu et al., 2020) sketched relevant ideas, which could be formalized in future research.

Finally, we note that the inferential framework we have developed here can be adapted to accommodate natural language data extracted, for example, from maintenance logs where engineers write about the faults they observe and the possible reasons for what they see. We can embed the causal reasoning behind the texts by mapping them onto the event tree and learning the structure of the causal CEG. This requires natural language processing techniques where the d‐events play a role as a bridge to link the tree to the free texts. Yu and Smith (2021b) and Yu (2021) proposed a naive way to implement this idea which used a hierarchical structure. These can be used to help structure appropriate CEGs like the examples as well as informing the posterior floret probabilities. More advanced algorithm can be developed in the future to automate this causal learning process.

## AUTHOR CONTRIBUTIONS

Development of the methodology behind the use of CEGs for modeling remedial maintenance regimes was led by Xuewen Yu with contributions by Jim Q. Smith; software and data analysis, Xuewen Yu; presentation of the material led by Xuewen Yu with contributions from Jim Q. Smith. Both authors have read and agreed to the published version of the manuscript.

## CONFLICT OF INTEREST STATEMENT

The authors declare no conflicts of interest.

## Supporting information

Figure 1: Error plots. Figure 2: Plot of failure life cycle of a system.

Data S1

## Footnotes

1 The idle CEG is the CEG that represents a system which is not intervened or manipulated.

2 The superscript indexes the order of the edge connecting $w_{1}$ to $w_{3}$ from top to bottom.

3 The R code implementing the analysis is available at https://github.com/xwyu2021. Other packages for CEGs are available, including R packages stagedtrees (Carli et al., 2020), ceg (Collazo & Taranti, 2017), and the python package cegpy (Walley et al., 2023).

4 Here, for simplicity, we do not consider symptoms.

5 Section 3 in the supplementary material provides a more detailed discussion on this point.
