# Supplementary material for: Causal chain event graphs for remedial maintenance
Source: Risk Anal. 2024 Apr 23;45(4):896–909. doi: 10.1111/risa.14308 (PMC12032386; doi:10.1111/risa.14308)
Supplement: Supplementary file 1 — Figure 1: Error plots. Figure 2: Plot of failure life cycle of a system. [file RISA-45-896-s001.pdf]

# SUPPLEMENT TO CAUSAL CHAIN EVENT GRAPHS FOR REMEDIAL MAINTENANCE

## 1 | THE PROOF OF THEOREM 3

If we can find a partition $\{\Lambda_z\}$ of $\Lambda(\boldsymbol{w}^*)$, then the singular intervention causal query conditional on $\Lambda(\boldsymbol{w}^*)$ can be written as:

$$\pi^{\Lambda(\boldsymbol{w}^*)}(\Lambda_y \| \Lambda_x) = \sum_z \pi^{\Lambda(\boldsymbol{w}^*)}(\Lambda_y | \hat{\Lambda}_x, \Lambda_z) \pi^{\Lambda(\boldsymbol{w}^*)}(\Lambda_z \| \Lambda_x). \tag{1}$$

If we estimate this intervened quantity from the partially observed system, then

$$\begin{aligned} \pi^{\Lambda(\boldsymbol{w}^*)}(\Lambda_y \| \Lambda_x) &= \sum_{\substack{w \in \boldsymbol{w}^* \\ e(w,w') \in e(x)}} \pi^{\Lambda(\boldsymbol{w}^*)}(\Lambda(w)) \pi^{\Lambda(\boldsymbol{w}^*)}(\Lambda_y | \Lambda(w')) \\ &= \sum_{\substack{w \in \boldsymbol{w}^* \\ e(w,w') \in e(x)}} \pi^{\Lambda(\boldsymbol{w}^*)}(\Lambda(w)) \pi^{\Lambda(\boldsymbol{w}^*)}(\Lambda_y | \Lambda(e_{w,w'})) \\ &= \sum_{\substack{w \in \boldsymbol{w}^* \\ e(w,w') \in e(x)}} \pi^{\Lambda(\boldsymbol{w}^*)}(\Lambda(w)) \sum_z \pi^{\Lambda(\boldsymbol{w}^*)}(\Lambda_y | \Lambda(e_{w,w'}), \Lambda_z) \pi^{\Lambda(\boldsymbol{w}^*)}(\Lambda_z | \Lambda(e_{w,w'})) \end{aligned} \tag{2}$$

By the two criteria specified in Theorem 2 for the back-door partition $\{\Lambda_z\}$, we can replace the last two terms in the last step by the following:

$$\begin{aligned} \pi^{\Lambda(\boldsymbol{w}^*)}(\Lambda_y \| \Lambda_x) &= \sum_{\substack{w \in \boldsymbol{w}^* \\ e(w,w') \in e(x)}} \pi^{\Lambda(\boldsymbol{w}^*)}(\Lambda(w)) \sum_z \pi^{\Lambda(\boldsymbol{w}^*)}(\Lambda_y | \Lambda(w), \Lambda_x, \Lambda_z) \pi^{\Lambda(\boldsymbol{w}^*)}(\Lambda_z | \Lambda(w)) \\ &= \sum_z \pi^{\Lambda(\boldsymbol{w}^*)}(\Lambda_y | \Lambda_x, \Lambda_z) \pi^{\Lambda(\boldsymbol{w}^*)}(\Lambda_z). \end{aligned} \tag{3}$$

Comparing with Equation (1), we therefore have the following two equivalent expressions.

$$\pi^{\Lambda(\boldsymbol{w}^*)}(\Lambda_y | \hat{\Lambda}_x, \Lambda_z) = \pi^{\Lambda(\boldsymbol{w}^*)}(\Lambda_y | \Lambda_x, \Lambda_z), \tag{4}$$

$$\pi^{\Lambda(\boldsymbol{w}^*)}(\Lambda_z \| \Lambda_x) = \pi^{\Lambda(\boldsymbol{w}^*)}(\Lambda_z). \tag{5}$$

## 2 | RESULTS OF THE SIMULATION STUDY

In Section 5, we used Dirichlet priors for the conditional probabilities $\boldsymbol{\theta}_v$. Collazo, Görgen, and Smith (2018) suggested treating each Dirichlet hyperparameter $\alpha_{v_i,v_j}$ as the number of phantom units arriving at the child node $v_j$ of $v_i$. Let $\alpha_0$ denote the number of phantom units entering the root node $v_0$ and weigh the edges emanating from the same node equally likely. For example, when $\alpha_0 = 1$, we have $\alpha_{v_0,v_1} = \alpha_{v_0,v_2} = 0.5$. We perform the analysis mentioned in Section 5 for different values of $\alpha_0$ and check the total situational error ($\varepsilon(\mathcal{T}) = \sum_{v \in V_{\mathcal{T}}} \|\boldsymbol{\theta}_v^{\dagger} - \tilde{\boldsymbol{\theta}}_v\|_2$). This error metric is the sum of the Euclidean distance between the true conditional probabilities $\boldsymbol{\theta}_v^{\dagger}$ and the mean posterior probabilities $\tilde{\boldsymbol{\theta}}_v$ estimated on the best scoring model for all stages. The results are depicted in Figure 1a, where we observe that the difference between the total situational errors for different $\alpha_0$ values is quite small. When decomposing the error for each stage, as shown in Figure 1b, the curves corresponding to different $\alpha_0$ values overlap. Thus the method is robust to the choice of hyperparameters. Other analyses or applications of the proposed method can be found in (Yu, Smith, & Nichols, 2020) and (Yu, 2021).

## 3 | INTERVENTIONS THAT IMPROVE SYSTEM EFFICIENCY

Figure 2 uses a bathtub curve to portray the life cycle of a unit, reflecting the change in failure rate. The perfect remedial intervention formalised in this paper corresponds to the black curve in the second life cycle. If the system is improved after

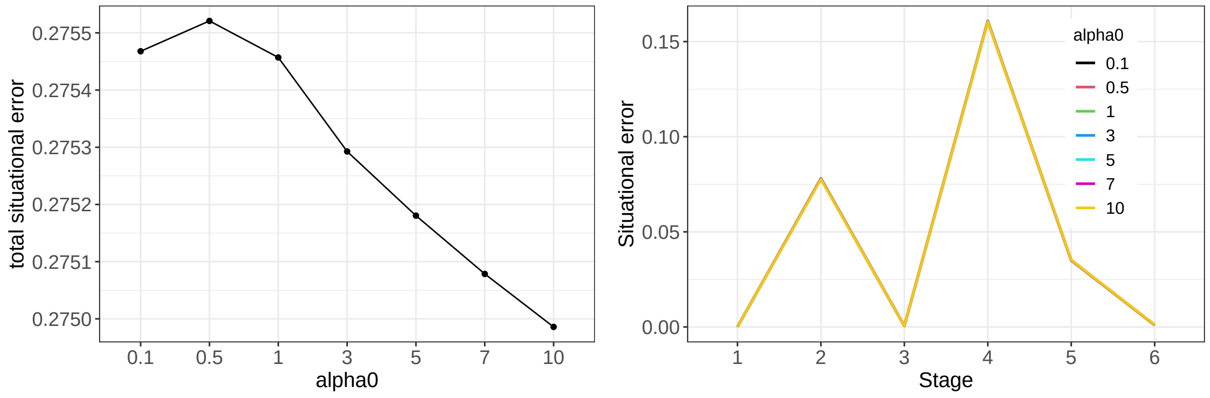


(a) Comparison of total errors for different values of $\alpha_0$.

(b) Comparison of situational errors for different $w_i$ values of $\alpha_0$.

**FIGURE 1** Error plots.

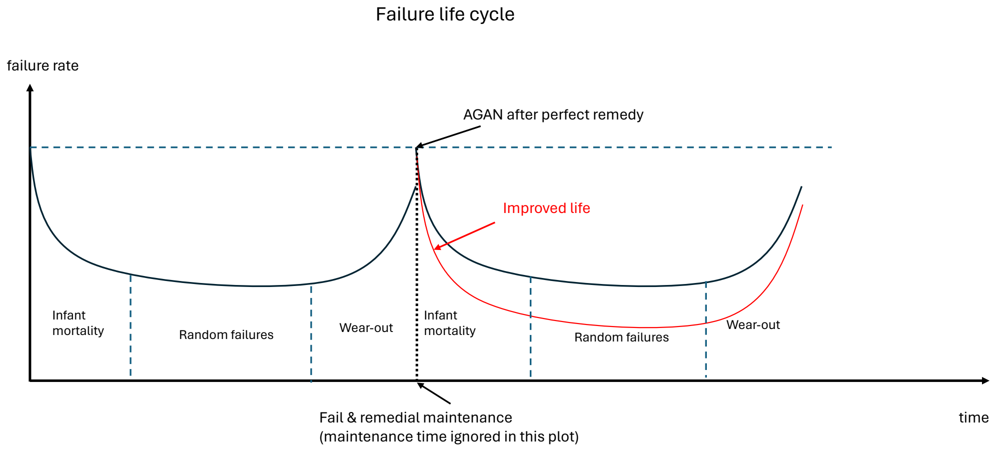


**FIGURE 2** Plot of failure life cycle of a system.

maintenance, then the second life cycle may look like the red curve where the failure rate is reduced throughout the entire lifetime of the unit compared to the first life cycle.

Such an intervention may involve manipulation not only of the probability distribution over root causes: either upstream or downstream florets of the root causes might be manipulated, depending on the maintenance. The algebras proposed in the paper can be generalised to encode such combinatorial manipulations. Moreover, distribution of time-to-event might also be affected. On a dynamic CEG, we can model semi-Markov processes with semi-Markov kernel $Q_{w_i,w_j}(t) = \theta_{w_i,w_j} P(h_{w_i,w_j} \leq t)$ where $h_{w_i,w_j}$ denotes the holding time at $w_i$ just before transitioning to $w_j$. We can specify a parametric distribution for each conditional holding time, for example, a Weibull distribution. Then controlling the shape parameter of the Weibull distribution enables us to work on different phases of the bathtub curve.

## References

Collazo, R. A., Görgen, C., & Smith, J. Q. (2018). *Chain event graphs*. CRC Press.

Yu, X. (2021). *Causal analysis on chain event graphs for reliability engineering* (Unpublished doctoral dissertation). University of Warwick.

Yu, X., Smith, J. Q., & Nichols, L. (2020). Bayesian learning of causal relationships for system reliability. *arXiv preprint arXiv:2002.06084*.
